# Clinical value of monoclonal antibodies and tyrosine kinase inhibitors in the treatment of head and neck squamous cell carcinoma

**DOI:** 10.1007/s12032-017-0918-1

**Published:** 2017-03-17

**Authors:** Wiktoria Blaszczak, Wojciech Barczak, Anna Wegner, Wojciech Golusinski, Wiktoria Maria Suchorska

**Affiliations:** 1 Radiobiology Lab, Department of Medical Physics, The Greater Poland Cancer Centre, Garbary 15 Str., 61-866 Poznan, Poland; 2 Department of Head and Neck Surgery, The Greater Poland Cancer Centre, Poznan University of Medical Sciences, Garbary 15 Str., 61-866 Poznan, Poland; 3 Department of Electroradiology, Poznan University of Medical Sciences, Garbary 15 Str., 61-866 Poznan, Poland

**Keywords:** Head and neck cancer, mAb, TKI, Therapy

## Abstract

Head and neck squamous cell carcinoma (HNSCC) is a heterogeneous group of malignant tumours that affects over 500,000 patients per year. Treatment failure is generally due to the heterogeneity of these tumours and to the serious adverse effects associated with treatment. Immunological system impairment, which is common in HNSCC, further contributes to treatment failure by mediating tumour escape mechanisms. To date, the only clinically approved targeted therapy agent is cetuximab, a monoclonal antibody (mAb) that binds to, and inhibits, epidermal growth factor receptor, which is widely overexpressed in HNSCC. Cetuximab has been proven to induce antibody-dependent cellular cytotoxicity, further magnifying its therapeutic effect. DNA sequencing of HNSCC cells has identified the presence of mutated genes, thus making their protein products potential targets for therapeutic inhibition. Immune mechanisms have been found to have a significant impact on carcinogenesis, thus providing the rationale to support efforts to identify anticancer compounds with immunomodulatory properties. In the context of the rapid development of novel targeted agents, the aim of the present paper is to review our current understanding of HNSCC and to review the novel anticancer agents (mAbs and TKIs) introduced in recent years, including an assessment of their efficacy and mechanisms of action.

## Introduction

Head and neck squamous cell carcinoma (HNSCC) is the sixth most common neoplasm worldwide, comprising a heterogeneous group of malignancies arising from the mucosal surfaces of the paranasal sinuses, the oral and nasal cavities, the pharynx, and the larynx [1]. Although our understanding of these tumours has improved significantly in recent years, treatment outcomes have barely improved [2]. The three most commonly reported risk factors for HNSCC are alcohol, tobacco, and human papilloma virus (HPV) infection [2]. Tobacco smoke has over 5000 chemicals, with at least 60 proven to be cytotoxic, mutagenic, and carcinogenic, which explains the important impact of tobacco on the incidence of HNSCC [3]. Tobacco smoke also increases the level of reactive oxygen species (ROS), which in turn stimulate expression of interleukin 8 (IL-8), a pro-inflammatory cytokine, leading to prolonged inflammation [4].

The most common treatments for head and neck cancer include surgery, radiation, and chemotherapy (CT), or a combination thereof. Currently, six agents have received Food and Drug Administration (FDA) approval for the treatment of HNSCC: cisplatin, 5-fluorouracil, docetaxel, methotrexate, bleomycin, and cetuximab, a monoclonal antibody (mAb). Platinum-based chemotherapy agents such as carboplatin and cisplatin, with an efficacy of up to 40%, are standard treatments for HNSCC, often used in combination with ionizing radiation [5]. Their mechanism of action is related to the formation of covalent bonds with nucleic acids. Docetaxel, which was approved by FDA in 2006 for use in locally advanced inoperable tumours, is a taxane that promotes cell cycle arrest and apoptosis [6]. Cisplatin is often used to treat non-resectable malignancies, metastatic lesions, and as a complementary chemotherapy agent. However, an important disadvantage of cytostatic agents is their lack of selectivity in targeting cells. Tumours are more susceptible to CT only because of their higher rate of division compared to healthy cells [6], which explains why CT is associated with high cytotoxicity and serious adverse effects including neutropenia, alopecia, stomatitis, and mucositis. These adverse effects are relevant because they can significantly decrease overall quality of life [7].

To date, cetuximab remains the only targeted agent for the treatment of HNSCC. Cetuximab was first proposed for use in HNSCC after it was discovered that epidermal growth factor receptor (EGFR) was significantly overexpressed in HNSCC and this overexpression is associated with worse prognosis [8] and greater radioresistance [9]. HNSCC tumours also significantly increase immunosuppression in patients, as evidenced by decreased absolute lymphocyte counts compared to healthy individuals [10]. Elevated levels of inflammatory cytokines (IL-6, TGF-β, VEGF, HGF) at the tumour site enhance cellular proliferation and migration [11, 12] and also increase the risk of relapse and metastasis [13]. Other characteristics observed in immunosuppressive HNSCC include impaired antigen-presenting functions [14], aberrant natural killer (NK) cell activity [15], and increased apoptosis of CD8^+^ cells [16]. In addition, dysregulation of antigen-presenting mechanisms is also typically present in HNSCC [14] and the impact of a dysregulated cytokine profile is crucial because tumours tend to favour immunosuppressive and pro-inflammatory cytokines rather than stimulatory cytokines, an imbalance that contributes to tumour immune escape mechanisms [11].

Given the rapid development of new agents with therapeutic potential for HNSCC, the aim of this paper is to review our current understanding of HNSCC and to assess the efficacy and mechanisms of action of the novel anticancer agents introduced in recent years.

## mAbs-based immunotherapy

Molecular heterogeneity in HNSCC significantly lowers the chance of identifying a single therapeutic agent that is effective for all HNSCC tumour types. However, sequencing analysis of HNSCC tumours has provided data to help identify numerous new potential therapeutic targets that are overexpressed in these carcinomas and believed to contribute to tumourigenesis [17, 18]. Such targets include the following: EGFR, vascular endothelial growth factor (VEGF), programmed cell death protein (PD-1), hepatocyte growth factor (HGF), phosphatidylinositol-4,5-bisphosphate 3-kinase (PI3K), c-Met pathway elements, cytotoxic T lymphocyte-associated protein 4 (CTLA-4), and CD137.

Cetuximab is a monoclonal chimeric antibody with the ability to bind to EGFR, a cell surface receptor of the EGFR tyrosine kinase inhibitor (TKI) family. The four other members of this family include EGFR1, HER2, HER3, and HER4 [4, 19]. Activated EGFR homo- or heterodimerizes along with other receptors result in phosphorylation of tyrosine and subsequent activation of signalling pathways such as MAPK, PI3K/Akt, or STAT. Induction of these pathways may result in dysregulation of apoptosis, proliferation, and transcription [11, 20].

Apart from targeting EGFR, cetuximab also stimulates the induction of antibody-dependent cellular cytotoxicity (ADCC). Recent studies have found that ADCC can be further enhanced by activating CD137, a co-stimulatory molecule overexpressed by NK and T cells after stimulation. CD137 expression in NK cells is triggered by exposure to cetuximab, thus increasing ADCC [21]. Based on this effect, it has been postulated that an additional application of CD137 agonists after cetuximab-based therapy might improve treatment response [22].

Kohrt et al. reported that cetuximab response is highly dependent on CD8^+^ cells. Enhancement of cetuximab response is mediated by dendritic cell maturation, induced by NK cells [23]. Furthermore, stimulation of peripheral blood cells through TLR 8^+^ (Toll-like receptor 8) further augments cetuximab-induced ADCC and promotes dendritic cell maturation in HNSCC [24].

However, HNSCC tumours can develop resistance to cetuximab. The mechanism behind this resistance process is signalling based, leading to activation of the other members of the EGFR family: HER2, HER3, as well as c-Met and insulin growth factor receptor (IGFR) [4, 19]. Another aim of therapeutic inhibition is activation of the c-Met/HGF signalling pathway, which is recognized as a major contributing factor to HNSCC resistance to EGFR, cisplatin, and radiation [25]. Antibodies targeting those pathways are under clinical evaluation and currently include transtuzumab, pertuzumab, onartuzumab, and cixutumumab [26, 27]. HGF also contributes to the inhibition of dendritic cell (DC) maturation. Anti-HGF mAb (AMG-102, rilotumumab) has been developed to eliminate this effect, and AMG-102 is currently undergoing evaluation in the treatment of renal cell carcinoma (RCC) [28], advanced solid tumours, and metastatic gastric esophagogastric adenocarcinoma [29]. Activation of c-Met can be blocked by ficlatuzumab, a humanized anti-HGF mAb currently being tested in combination with cetuximab in HNSCC [30]. Increased expression of c-Met correlates with resistance to platinum-based agents, radiation, and to EGFR-targeting agents; given that most HNSCC samples overexpress c-Met, this may be a novel therapeutic target [31].

Hayakawa et al. [32] found that IL-6 levels were upregulated in HNSCC, and elevated levels of IL-6 are associated with STAT3 signalling and blockage of DC maturation, which makes them a potential target for therapeutic inhibition. Siltuximab, a chimeric monoclonal antibody directed against IL-6, was designed to restore physiological STAT3 signalling and DC maturation. The efficacy of siltuximab in the treatment of metastatic prostate cancer is under evaluation [33].

Another EGFR-targeting antibody is zalutumumab [34]. This human IgG1 mAb is characterized by high affinity to EGFR and low immunogenicity. The main advantage of zalutumumab is its immunogenicity, which, while improving overall tolerance, does not impair ADCC induction [35]. The efficacy of zalutumumab has been evaluated in incurable HNSCC, where it increased overall survival by 1.5 months versus healthy controls [36]. Panitumumab, another anti-EGFR molecule, is a human IgG2 mAb with less capacity to enhance ADCC compared to cetuximab. Panitumumab, which has a reduced affinity for the CD16 receptor, has been shown to present fewer hypersensitivity reactions. Panitumumab has been evaluated in combination with cisplatin and 5-FU in HNSCC, yielding a greater than threefold decrease in disease progression when compared to CT alone; however, this combined treatment was also associated with a significant increase in serious adverse effects [37]. The efficacy of panitumumab in monotherapy [38] is currently being evaluated [20]. Nimotuzumab is a recombinant humanized murine antibody targeting EGFR. The activity of nimotuzumab as a complement to standard chemotherapy has been evaluated in locally advanced HNSCC [39]; assessment of its efficacy combined with CRT is ongoing [40].

MEHD7945A is another anticancer mAb whose main advantage is an affinity for both EGFR and HER3 [41]. MEHD7945A may act as a radiation sensitizer in both lung cancer and HNSCC. Studies conducted to date have shown that this drug can effectively enhance radiation response in those neoplasms, both in vitro and in vivo [41].

Angiogenesis is crucial to tumour proliferation, invasion, and metastasis. The process is enhanced by low oxygen levels and VEGF overexpression [42]. Considering the importance of VEGF in inducing vascularization, elevated levels of VEGF (both the growth factor and its receptors) correlate with poor prognosis [24], which explains the interest in bevacizumab, a VEGF-targeted mAb. The main advantage of bevacizumab is its affinity for all five VEGF isophorms [43]. However, studies that have investigated bevacizumab as monotherapy have found no significant change in response or overall survival [44]. By contrast, when it is added as a complementary treatment to standard, platinum-based CT, it substantially improves the outcomes of patients with advanced non-small cell lung carcinoma (NSCLC) [43]. This raises the possibility that bevacizumab could have a similar effect in HNSCC [44]. A trial of bevacizumab with CT in locally advanced HNSCC is currently ongoing [45].

The clinical use of mAbs such as cetuximab and trastuzumab (which targets EGFR and HER2), designed to inhibit signalling promoting cell proliferation and evasion of apoptosis, yields a better overall response than TKIs targeting the same molecules [11]. Those findings suggest that some other immune mechanisms, apart from blockage of downstream signalling, must contribute to the clinical success of these agents [11]. In xenograft models expressing HER2, trastuzumab and pertuzumab induced ADCC [46], leading the authors to suggest that co-targeting tumour antigens (TA) with mAbs might further enhance immune response.

The loss of phosphatase and tensin homologue (PTEN) and transforming growth factor β receptor 1 (TGF-βR1) are associated with PD-L1 activation, possibly through the Akt signalling pathway [47, 48]. Studies have found PD-1 overexpression in 68–70% of HNSCC malignancies [26], regardless of HPV infection status [49, 50]. PD-1 is a receptor present on the surface of cytotoxic T lymphocytes (CTLs), B and NK cells, and macrophages. PD-1 is expected to be superior to CTLA-4 in negative signalling. PD-L1 and PD-L2 are ligands of PD-1. After prolonged exposure to antigens, macrophages and dendritic cells start to overexpress PD-L1, which, in the HNSCC setting, leads to decreased CTL and NK cell activity [51]. If antigen stimulation continues, CTLA-4 and PD-1 receptors downregulate CTL response, thus resolving post-infectious inflammation and thwarting the autoimmune response. Those “immune checkpoints” serve a useful purpose in cancers; however, they may lead to pathologic tolerance and thus contribute to tumour immune escape [48]. A humanized IgG4 anti-PD-1 mAb (nivolumab) has been developed to release CTLs and NK cells from anergy, and this drug has been evaluated in renal carcinoma, melanoma, and NSCLC. The evaluation of nivolumab in HNSCC is ongoing [52]. Pembrolizumab, an anti-PD1 mAb, is currently in phase 2 clinical trial in patients with metastatic HNSCC after standard platinum-based therapy [53]. Durvalumab also targets PD-L1, however is less effective than pembrolizumab, and evokes approximately 11% response [54].

The development of agents to reduce co-inhibitory signalling and activate CTLs is a recent aim of HNSCC treatment and ipilimumab is one of the agents that is potentially capable of achieving this effect. This anti-CTLA-4 IgG1 mAb is currently used to treat melanoma [55]. A decrease in co-inhibitory CTLA-4 signalling leads to amplification of tumour-specific CTLs, thus having a therapeutic effect [56]. However, such agents should be administered with caution since they have been reported to cause severe adverse effects such as hypopituitarism or colitis [57]. Another agent with a similar mechanism in current trials is tremelimumab [58].

mAbs targeting PD-1 and PDL-1 have shown a significant response in solid tumours such as melanoma, renal cell carcinoma, and NSCLC [11]. Recent studies indicate that application of anti-PD-1 mAb in HNSCC significantly inhibits proliferation and decreases levels of myeloid-derived suppressor cells (MDSCs) and tumour-associated macrophages (TAMs), while elevating CD8^+^ cell population levels [49]. Nivolumab, an anti-PD-1 mAb, has been shown to have lower toxicity than CTLA-4-targeted antibodies; moreover, the adverse effects associated with therapy tend to be less severe and include fatigue and pyrexia [46]. Currently, nivolumab is in phase III trial in metastatic HNSCC [59].

OX40 (a tumour necrosis factor receptor) is a component of the co-stimulatory pathway, which can strengthen T cell memory and anti-tumour activity, thus reducing cancer immune escape mechanisms [60]. Main reason to use OX40 is that it has a proven co-expression with PD-1 and CTLA-4 [48]. Antibodies targeting OX40 might increase T cell signalling and enhance the proliferation of these T cells, memory, and cytotoxicity. Several studies have found that OX40-targeting mAbs may have synergistic effects when administered with other immunotherapeutics [61]. Guo et al. have shown that the combination of anti-OX40 mAb and anti-PD-1 improves overall survival compared to any of these agents alone [62, 63].

## Tyrosine kinase inhibitors (TKIs)

Another group of agents that has emerged recently are TKIs. These are a class of chemotherapeutics that block specific tyrosine kinases involved in pathways essential for tumour growth, invasion, and metastasis. By binding to the tyrosine kinase domain of EGFR, TKIs block subsequent signalling, resulting in inhibition of cell proliferation [62].

Two most commonly used TKIs are gefitinib and erlotinib. Gefitinib, which targets EGFR, causes reversible inhibition of the receptor; this TKI has been tested in conjunction with standard CT or CRT in HNSCC, and in 2015, it was approved by the FDA for monotherapy treatment of NSCLC. Erlotinib is another EGFR kinase inhibitor whose efficacy has been proven in over 80 clinical trials. It is currently approved for NSCLC and pancreatic cancer. Although trials have shown that erlotinib yields good results when added to radiotherapy, its use in combination with cisplatin is not recommended. A phase III clinical trial of erlotinib–cisplatin–radiation efficacy is ongoing [64].

Since heterodimerization of EGFR with other receptors is suspected to limit the benefits of mAbs and TKIs, it has been suggested that agents that can simultaneously inhibit several members of the EGFR family could be useful [62]. This explains the growing interest in small molecule inhibitors—such as lapatinib, afatinib, sorafenib, and sunitinib—that target multiple receptors. Lapatinib is a reversible TKI, directed against both EGFR and HER2 kinases. Afatinib, through covalent bond formation, irreversibly blocks EGFR, HER2, and HER4 kinases. One trial showed that co-administration of afatinib with cetuximab in HNSCC significantly reduced cell viability, suggesting an additive or synergistic effect [65]. The efficacy of sunitinib and sorafenib is due to inhibition of VEGFR, PDGFR, Flt3, and c-kit.

The hallmark of HNSCC tumour cells is an imbalance in STAT1/STAT3 signalling. While downregulation of the STAT1 signalling pathway decreases the concentration of CCL5 and CXCL10 (cytokines that promote T cell migration), upregulation of STAT3 increases cytokine secretion. IL-6, IL-10, TGF-β1, and VEGF contribute to impaired DC maturation and NK cell-mediated cytolysis [66]. The function of IFN-γ is to enhance HLA and antigen presentation in tumour and dendritic cells. This process is downregulated in the tumour environment due to lack of IFNγ-induced STAT1 signalling. Moreover, DC maturation is inhibited by the downregulation of the STAT3 pathway mediated by secretion of IL-6 by tumour cells. The therapeutic aim is to restore the balance between the STAT1/STAT3 pathways, either by inhibiting enhanced STAT3 or by inducing suppressed STAT1. In vitro studies have shown that downregulation of STAT3 signalling reduces the immunosuppressive characteristics of HNSCC [67]. An anti-Src TKI (dasatinib) may be useful due to STAT3 mediation execution. Two clinical trials have evaluated the efficacy of dasatinib in combination with cetuximab and CT [68, 69].

The use of Janus kinases offers another approach to reverse the STAT1/STAT3 imbalance. STAT3 is activated after interaction with JAK1, and JAK2 and Src. JAK1 and JAK2 are phosphorylated through IL-6-mediated stimulation. For this reason, researchers have recently sought to develop agents to downregulate STAT3 by targeting JAK receptors. Research in this area has led to the development of the novel drug ruxolitinib. JAK1 and JAK2 inhibitors have been approved for the treatment of myeloproliferative neoplasms [70]. A second JAK1 and JAK2 inhibitor—AZD1480—has been tested both in vitro and in vivo, with findings showing that AZD1480 effectively decreases IL-6-mediated STAT3 activation, thus inhibiting cell proliferation and inducing apoptosis. In mice, administration of AZD1480 has been reported to increase overall survival [71].

PI3Ks (phosphatidylinositide 3-kinases) are a family of enzymes engaged in various cellular processes such as proliferation, differentiation, and motility, which are related to tumourigenesis. The PIK3A gene is often mutated in HNSCC, making the pathway an attractive target for therapeutic inhibition. Furthermore, mutations in PIK3A elements are responsible for the subsequent activation of the Akt and mTOR pathways. To date, several small molecule inhibitors of PI3K have been developed, including GDC-0941, PX-866, NVP-BKM120, and NVPBYL719.

NK cells are one of the most important components of innate immunity, and these large cytotoxic lymphocytes are the main drivers of ADCC execution [72]. However, their activity in HNSCC may be impaired due to downregulation of the NK cell receptor (NKG2D). NKG2D depletion is promoted by increased TGF-β levels [51]. For this reason, it has been suggested that immunotherapy could improve response provided that expression of TGF-β concentrations at the tumour site can be decreased; one approach to reducing TGF-β expression is through c-Met blockage, which lowers the production of TGF-β, thus sensitizing tumour cells to immune mechanisms. Kumai et al. have shown that tivantinib, a TKI targeting c-Met, leads to a significant decrease in TGF-β production, thus resulting in better response and improved immune mechanisms. Collectively, previous studies have found a critical role for the c-Met/HGF pathway in cancer proliferation and metastasis, indicating that the c-Met/HGF may be a viable target for future therapy development [73]. Table 1 shows a compilation of the novel targeted therapy agents that may be useful in HNSCC treatment.

**Table 1:** Compilation of the novel targeted therapy agents in HNSCC treatment

Drug	Mechanism	Target	Phase	Status	Sponsor	References
Cetuximab	mAb	EGFR		FDA approved	ImClone Systems, Inc.	
Panitumumab		EGFR	II	Ongoing	Amgen	[38]
Nimotuzumab		EGFR	II	Ongoing	National Cancer Centre	[40]
Zalutumumab		EGFR	III	Completed	Genmab	[36]
MEDH7945A		EGFR, HER3	II	Completed	Genentech	[74]
Transtuzumab		HER2	II	Unknown	Bristol-Myers Squibb, Genentech	[75]
AV-203		HER3	I	Completed	AVEO Pharmaceuticals, Inc.	[76]
Cixutumumab		IGFR	II	Completed	ImClone LLC	[77]
Bevacizumab		VEGF	II	Ongoing	Woondong Jeong	[45]
Pembrolizumab		PD-1	II	Ongoing	Merck Sharp and Dohme Corp	[53]
Nivolumab		PD-1	III	Ongoing	Bristol-Myers Squibb	[59]
Durvalumab		PD-L1	II	Recruiting	AstraZeneca	[54]
Onartuzumab		c-Met	II	Completed	Genentech, Inc.	[78]
Rilotumumab		HGF	II	Completed	Amgen	[28]
Ficlatuzumab		HGF	I	Recruiting	Julie E. Bauman, MD, MPH	[30]
Siltuximab		IL-6	II	Completed	Southwest Oncology Group	[33]
Ipilimumab		CTLA-4	I	Recruiting	National Cancer Institute	[79]
Tremelimumab		CTLA-4	III	Recruiting	AstraZeneca	[58]
Urelumab		CD137	I	Ongoing	Bristol-Myers Squibb	[80]
Gefitinib	TKI	EGFR	II	Completed	AstraZeneca	[81]
Erlotinib		EGFR	III	Ongoing	Grupo de Investigación Clínica en Oncología Radioterapia	[64]
Dacomitinib		EGFR	I,II	Completed	University Health Network	[82]
Lapatinib		EGFR, HER2	III	Completed	GlaxoSmithKline	[83]
Afatinib		EGFR, HER2, HER4	III	Recruiting	Centre Leon Berard	[84]
Sunitinib		VEGFR, PDGFR, Flt3, c-kit	I	Terminated	National Cancer Institute	[85]
AZD-1480		JAK1, 2	I	Terminated	AstraZeneca	[86]
Ruxolitinib		JAK1, 2	0	Not yet recruiting	University of Pittsburgh	[87]
Tivantinib		c-Met	II	Ongoing	National Cancer Institute	[88]
Foretinib		VEGFR, c-Met	II	Completed	GlaxoSmithKline	[89]
Sorafenib		VEGFR, PDGFR, Raf	I, II	Completed	Duke University	[90]
Dasatinib		Src	I, II	Ongoing	Sidney Kimmel Comprehensive Cancer Center	[69]
GDC-0941		PI3K	I	Completed	Genentech, Inc.	[91]
PX-866		PI3 K	I, II	Completed	Oncothyreon Inc.	[92]
NVP-BKM120		PI3K	II	Ongoing	Novartis Pharmaceuticals	[93]
PVP BYL719		PI3K	II	Recruiting	Novartis Pharmaceuticals	[94]
Everolimus		mTOR	I	Not yet open	M.D. Anderson Cancer Center	[95]
IL-2	Immunomodulators		II	Completed	H. Lee Moffitt Cancer Center and Research Institute	[96]
IL-12			I, II	Ongoing	National Cancer Institute	[97]
IFN-α2a			III	Completed	Eastern Cooperative Oncology Group	[98]
Bortezomib	Proteasome		II	Completed	Vanderbilt-Ingram Cancer Center	[99]

## Conclusions

Immune response is crucial in most cancers, particularly in HNSCC, because the tumour response may contribute to tumour evasion mechanisms, resistance to therapeutic agents, and disease progression. At present, the main aim of immunotherapy in HNSCC is to magnify the immune response, such as ADCC, and to sensitize cells to conventional treatments. Compared to standard treatments, the main advantage of immunotherapy is its distinctly higher specificity, which implies less cytotoxicity and better overall tolerance. The immunosuppressive characteristics of HNSCC stem from elevated levels of inflammatory cytokines in the tumour microenvironment. This, apart from mediating cellular proliferation and migration, also contributes to higher relapse rates and metastasis. Therefore, restoring balance to the cytokine profile could yield treatment benefits. The search for novel agents will undoubtedly continue, and it is essential that we determine the most effective combinations of known therapeutic compounds in order to improve treatment outcomes in HNSCC.
